# Relationships between Renewable Energy and the Prevalence of Morbidity in the Countries of the European Union: A Panel Regression Approach

**DOI:** 10.3390/ijerph18126548

**Published:** 2021-06-18

**Authors:** Robert Stefko, Beata Gavurova, Miroslav Kelemen, Martin Rigelsky, Viera Ivankova

**Affiliations:** 1 Faculty of Management, University of Prešov in Prešov, Konštantínova 16, 080 01 Prešov, Slovakia; robert.stefko@unipo.sk (R.S.); martin.rigelsky@smail.unipo.sk (M.R.); viera.ivankova@smail.unipo.sk (V.I.); 2 Center for Applied Economic Research, Faculty of Management and Economics, Tomas Bata University in Zlín, Mostní 5139, 760 00 Zlín, Czech Republic; 3 Faculty of Aeronautics, Technical University of Kosice, 041 21 Kosice, Slovakia; miroslav.kelemen@tuke.sk

**Keywords:** renewable energy, transport, electricity, heating and cooling, prevalence, epidemiology, public health, panel regression, fixed effects model, random effects model, European Union

## Abstract

The main objective of the presented study was to examine the associations between the use of renewable energy sources in selected sectors (transport, electricity, heating, and cooling) and the prevalence of selected groups of diseases in the European Union, with an emphasis on the application of statistical methods considering the structure of data. The analyses included data on 27 countries of the European Union from 2010 to 2019 published in the Eurostat database and the Global Burden of Disease Study. Panel regression models (pooling model, fixed (within) effects model, random effects model) were primarily used in analytical procedures, in which a panel variable was represented by countries. In most cases, positive and significant associations between the use of renewable energy sources and the prevalence of diseases were confirmed. The results of panel regression models could be generally interpreted as meaning that renewable energy sources are associated with the prevalence of diseases such as cardiovascular diseases, diabetes and kidney diseases, digestive diseases, musculoskeletal disorders, neoplasms, sense organ diseases, and skin and subcutaneous diseases at a significance level (α) of 0.05 and lower. These findings could be explained by the awareness of the health problem and the response in the form of preference for renewable energy sources. Regarding statistical methods used for country data or for data with a specific structure, it is recommended to use the methods that take this structure into account. The absence of these methods could lead to misleading conclusions.

## 1. Introduction

Energy is a fundamental necessity of modern life, but its dark side is the fact that the energy sector is responsible for more than 75% of greenhouse gas emissions in the European Union [1]. Today, there is a need to focus on renewable energy technologies that have the potential to improve the environment in terms of reducing greenhouse gases and global warming [2], which can also affect human health [3,4,5]. Climate change and global warming may lead to a significant increase in heat-related mortality and morbidity in the future [5]. In this context, renewable energy appears to be a key aspect to improve the environment and health, but renewable energy also has a positive effect on economic growth and human development [6,7]. Based on these benefits, renewable energy plays an important role in a modern and responsible world consisting of healthy and prosperous countries. Increasing the use of energy from renewable sources in various sectors of the economy is therefore a key element of an integrated energy system aimed to achieve climate neutrality as the main environmental ambition of the countries of the European Union [1,8]. In this sense, many innovative ideas offer positive prospects for improving the situation [9], while the basic pillar of the solution is the use of solar power, wind power, ocean and hydropower, biomass, and others [10].

The growing emphasis on proactive action on the part of developed countries cannot be overlooked. In particular, renewable energy policies in the European Union, but also research and scientific dissemination, are key to achieving the goals, and should take into account all forms of incentives that are offered to them [11]. In 2018, the 27 countries of the European Union achieved 18.9% of energy from renewable sources in total energy consumption, while their goal is to increase this share in further years [12]. In this sense, the share of energy from renewable sources in the electricity, heating, and cooling sectors was systematically above the level of the expected increasing trajectory, but in the transport sector it was slightly below the share planned by the National Renewable Energy Action Plans for the Member States of the European Union [13].

The evidence revealed by a newly developed technique, dynamic panel analysis under cross-sectional dependence, clearly shows that renewable energy causes a reduction in environmental degradation [14]. At the same time, it is well-known that environmental degradation and pollution have a negative effect on public health [15]. Therefore, it seems to be clear that there is a link between renewable energy and health.

Findings for a panel of 42 African countries between 1995 and 2011 revealed a long-run unidirectional causality running from renewable energy to health expenditure as an indicator of health [16], suggesting the importance of this issue for public health as such. The authors dealing with this topic, Apergis et al. [16], used a number of methodologies relevant to panel data to verify interactions, namely second-generation panel unit root tests, panel cointegration approaches, panel long-run estimates, and panel causality tests. Their results can be explained by the fact that if countries used their renewable sources efficiently, the benefits would come from reduced fossil energy bills and air pollution levels, and this would enable countries to save money for health care and, subsequently, to improve the health status of the population. The authors also emphasized the implementation of modern renewable energy projects in the health care sector [16]. Very similar findings were provided in a study conducted by Mujtaba and Shahzad [17], who addressed this issue in 28 countries of the Organisation for Economic Co-operation and Development (OECD) between 2002 and 2018. In their study, a panel fully modified ordinary least squares (OLS) regression model and cointegration tests were applied, while the authors confirmed long-run causality from renewable energy and carbon dioxide emissions to health care expenditure, as well as a significant and positive association between renewable energy and health care expenditure [17]. With a focus on another health indicator, Ben Jebli [18] analysed the relationship between the consumption of combustible renewables and waste and health status expressed by a number of doctors. Using the autoregressive distributed lag approach, the author [18] found that combustible renewables and waste consumption have a positive and significant effect on health, where simultaneously, the estimated lagged error correction terms showed a bidirectional long-run causality between health and combustible renewable waste consumption.

The findings in the previous paragraph indicate a possible relationship between renewable energy and health as such, that is, health expressed by morbidity or mortality. As it has been shown that the use of renewable energy can translate into higher health care expenditure and a higher number of doctors, an improvement in the health of the population can be expected.

Another study was conducted by Taghizadeh-Hesary et al. [19], who examined the relationship between non-renewable energy sources and health, and they used a generalized method of moments (GMM) estimation technique as a panel data analysis for 18 low- and middle-income countries between 1991 and 2018. Their results provided evidence that fossil fuel energy consumption increases the risk of lung and respiratory diseases, and there was a significant effect of carbon dioxide emissions and fossil fuel consumption on undernourishment and mortality rates. Similar results were revealed by Khan et al. [20], who used the techniques of fully generalized least squares (FGLS) and GMM estimation in a sample of 10 Central European countries over the period 1991–2018. As the consumption of non-renewable energy sources may lead to greater environmental degradation and pollution with a negative effect on public health, it is recommended to focus more intensively on renewable energy sources, which could improve the situation of energy insecurity, reduce greenhouse gas emissions, and decrease negative health effects [19,20,21]. In other words, energy efficiency and renewable energy can be beneficial for the environment and public health [22,23]. However, there are still many opportunities to examine the issue from different perspectives and using different statistical methods.

There are also many statistical methods that can be used in the examined issue. Regression analysis is a basic tool, but it is still used in different variations in contemporary studies with a transnational effect. Regression models are commonly applied to cross-sectional or time series data, while a panel regression model is some kind of compromise. The advantage of the panel regression models is evident especially in its ability to identify and take into account effects that cannot be found by cross-sectional models, where it means that the panel regression models take the data structure into account. Another equally important advantage is the ability to control individual heterogeneity, to increase variability for more efficient estimation or to increase the accuracy of estimates, as these models work with microdata and not aggregated data [24]. Currently, the panel models represent a relatively large part of the statistical investigation. There are not only classical one-way and two-ways variations of fixed effects models, random effects models, and nested models, but also well-known strong dynamic models, models capable of solving endogeneity problems, count data models, or spatial models [25]. The use of these models has found wide application possibilities especially in econometrics or public health, as when using high-quality statistical methods, it is possible to achieve very high-quality and relevant results. An important part of proper use is deciding on the choice of a specific model, taking into account several assumptions. Among the most commonly used tests for this purpose are the Breusch-Pagan test [26] that helps to assess the variability of residues, Wooldridge’s test for unobserved individual effects [27] that assesses the significance of unobservable effects through residue distribution, the Baltagi and Li one-sided LM test [28] that assesses the significance of the internal data structure and thus the suitability of the use of panel models, and likewise, the F test for individual and/or time effects. In addition, there is the Hausman test and its robust variant, which help to decide on a model with fixed (within) effects or a model as a Generalized Least Squares (GLS) alternative in the form of random effects. It is also possible to mention Angrist and Newey’s test [29], which identifies the limitations of models with fixed effects.

The presented research studies examining the connection of the renewable energy dimension with the health dimension are quite heterogeneous, but they provide valuable information about the applied methods, their application potential, and limitations. Thus, they enable the creation of an area for subsequent research and to formulate new research trajectories. The development of a methodological platform in each research area is quite demanding as it is a dynamic process, while it is extremely important to create international research networks to share knowledge from the application of the methodological processes. Additionally, the development of social systems, the processes of globalisation, and demographic development are important determinants influencing the methodological processes that are also related to the difficult nature of the data [30,31]. For this reason, an issue of examination of the applicability of the methods and the methodological processes linking different research areas, such as renewable resources and health, possesses great importance [32,33,34]. The creation of the national and international policies is determined by the availability of the quality research reports that have to not only aggregate the current situation, but also reveal the causes of this situation and the possibilities of its solution to quantify the effect of the alternatives, and thus, to help to create stabilisation and the regulatory mechanisms [35,36,37]. Without the appropriate analyses, it is not possible to create the relevant policies, but these analyses also require access to the deeper structured data enabling the emergence of the new methodological procedures, as well as the development of the current ones [38,39,40,41]. These consistent facts created motivation for us to carry out our research aimed at examination of the selected dimensions of the use of renewable energy resources and the health parameters—the prevalence of the selected diseases.

## 2. Materials and Methods

The main objective of the presented study was to examine the associations between the use of renewable energy sources in selected sectors (transport, electricity, and heating and cooling) and the prevalence of selected groups of diseases in the European Union, with an emphasis on the application of statistical methods considering the structure of data. The classification of sectors, namely transport, electricity, and heating and cooling, represents one of the structures of renewable energy consumption to total energy consumption in the Eurostat database. This classification was chosen on the basis of the most appropriate logical connection with the application aspect. To achieve our objectives, several analytical procedures were performed, the most important of which was regression analysis. This analysis was implemented in three variants (pooling model, fixed effects model, random effects model) and the selection of one of them was conditioned by individual tests of assumptions. When using the analyses, the emphasis was placed on the need to take the structure of data into account (in this case, the structure of countries).

In the context of achieving the objective of the study, three research questions were formulated, which also determine the methodological framework of the study within three research areas:

RQ1: Is there an association between the share of energy from renewable sources in total energy consumption in the transport sector and the prevalence of diseases classified into selected diagnosis groups?

RQ2: Is there an association between the share of energy from renewable sources in total energy consumption in the electricity sector and the prevalence of diseases classified into selected diagnosis groups?

RQ3: Is there an association between the share of energy from renewable sources in total energy consumption in the heating and cooling sector and the prevalence of diseases classified into selected diagnosis groups?

The analytical procedures included data from the Eurostat database [42], namely the share of energy from renewable sources as an environmental indicator, and data from the Global Burden of Disease Study [43], specifically, health indicators of disease prevalence. The data were collected for the period 2010–2019. Thus, each of the countries of the European Union reported annual data for the observed period, that is, 10 years for individual variables. It should also be noted that no missing data were found.

The share of energy from renewable sources in total energy consumption (RNWe) appeared in the classification of three sectors: (i) transport (RNWe TSP), (ii) electricity (RNWe ELC), and (iii) heating and cooling (RNWe H&C). This environmental indicator was presented as a percentage of renewable energy sources from total consumption, while the higher the value, the higher the consumption of renewable sources.

The diagnosis groups covered 11 areas of diseases: cardiovascular diseases (CRD), diabetes and kidney diseases (DIA), digestive diseases (DGS), chronic respiratory diseases (RSP), mental disorders (MNT), musculoskeletal disorders (MLT), neoplasms (NPL), neurological disorders (NRL), sense organ diseases (SNS), skin and subcutaneous diseases (SKN), and substance use disorders (SBC). The values of these health indicators represented the prevalence of diseases calculated per 100,000 inhabitants of individual countries.

In general, this study focused on the analysis of the associations between the change in the share of energy from renewable sources in total energy consumption (in%) and the change in the prevalence of diseases classified into selected diagnosis groups (prevalence per 100,000 population in a country).

Several statistical procedures were selected for analytical processing. First, a statistical description and additional visualizations showing trends in selected variables were provided for a more detailed look at the indicators. Non-parametric tests of differences (Kruskal Wallis test) were also used, which were preferred based on the results of the Shapiro–Wilk test of normality. Second, the assumptions were assessed in order to choose a suitable panel regression model. The Baltagi and Li one-sided LM test was chosen to identify the possible occurrence of a serial correlation [28]. The F test for the presence of individual effects (or time effects) was used to assess the significance of effects in the internal data structure in terms of individual countries, but also individual years. The robust regression-based Hausman test (vcov: vcovHC) was used in order to appropriately choose a fixed (within) effects model or a random effects model. Third, the effects were presented in three variants of regression models, including a robust version of the OLS pooling model (vcovHC), a model with fixed effects, specifically the one-way (individual) effects within model (Arellano estimator), and a model with random effects, specifically the one-way (individual) random effect model: Swamy–Arora’s transformation (White 2 estimator).

The essence of the above-mentioned tests lies in the optimal selection of a particular regression (panel) model. If the Baltagi and Li one-sided LM test showed a significant result, a robust estimator was preferred. If the F test showed a significant result, such as for countries, the internal data structure from the point of view of countries was taken into account, and a one-way model was preferred. If the F test showed a significant result both for countries and for time, it would be appropriate to prefer a two-way model. Commonly used panel models were applied, namely the fixed (within) effects model and the random effects model. The choice of a suitable alternative between the models was supported by the Hausman test, in which a significant result suggests the use of the fixed (within) effects model.

The main analytical calculations were performed using the programming language R v 4.0.3 (RStudio, Inc., Boston, MA, USA), while Tableau v 2020.2 (Tableau Soft-ware, LLC, Seattle, WA, USA) was used secondarily.

## 3. Results

This section presents the results of analytical procedures, which were divided into two parts according to the used analyses: (i) descriptive analysis and (ii) regression analysis. The Appendix A contains a table showing the average values of the processed variables.

Table 1 provides the basic output of descriptive statistics, and attention should be paid to the measures of central tendency (average, median). In terms of the share of energy from renewable sources, the lowest share was found in the transport sector (RNWe TSP mean = 6.00; median = 5.67) and the other two sectors were roughly balanced (mean: RNWe ELC = 27.18; RNWe HaC = 26.35). With a focus on the health variables, neurological disorders (mean NRL = 42,982.91) could be considered the diagnosis group with the highest prevalence, while the lowest mean prevalence was shown in substance use disorders (mean SBC = 3262.79). At this point, it should be noted that the chosen metric (prevalence per 100,000 population) does not take into account the severity of the disease, and focuses only on the occurrence of specific diagnoses of the group. When assessing the descriptive measures, it should be borne in mind that these outcomes have been obtained by the countries of the European Union over a period of time. The skewness and kurtosis measures showed possible deviations, and the highest deviation from the normal distribution can be observed in the variable RNWe TSP (Skew = 2.48, Kurt = 8.99).

Table 2 shows the results of the univariate Shapiro-Wilk normality test (U SW), as well as the non-parametric Kruskal–Wallis tests to identify statistically significant differences in selected indicators between countries (KW C) and between years (KW Y). Based on the above-mentioned results, it could be stated that the assumption of normality was not confirmed for the vast majority of variables. The preference for non-parametric tests was therefore more acceptable. The results of the tests of differences between countries showed significant values in all cases. Accordingly, it was possible to confirm significant differences in selected environmental and health indicators between the analysed countries. However, on the basis of this test, it was not possible to identify the countries between which the differences were found and between which no differences were found. Thus, Appendix A shows the average values in the classification of countries, which may provide a closer look at the results. Differences within the classification of years were significant only in two cases (RNWe TRP = 53.52 †; DIA = 23.30 ***). These results indicated that when examining the relationships between the indicators, it was appropriate to use methods that are able to take the structure of countries into account.

**Figure 1:**
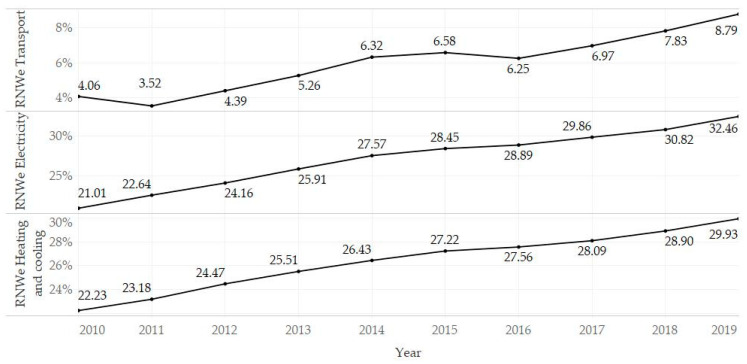
Development of the share of energy from renewable sources in total energy consumption (%) in the observed period (2010–2019).

**Figure 2:**
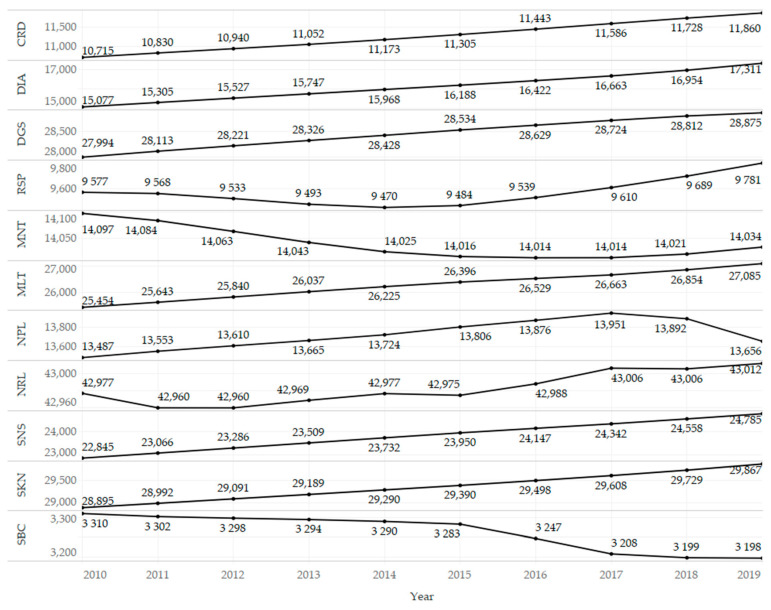
Development of the prevalence of diseases classified into selected diagnosis groups (per population of 100,000) in the observed period (2010–2019).

Figure 1 shows the development of the share of energy from renewable sources, and an upward trend is evident. The year-on-year changes were slightly unstable in the transport sector compared to the electricity and heating and cooling sectors, where the growth rate was relatively stable. Figure 2 presents the development of the prevalence of diseases classified into selected diagnosis groups, and an upward trend could be observed in most cases. This can be explained by population growth, increasing life expectancy, improving diagnostic methods in health care, but also by a deteriorating environment, when the use of renewable energy sources needs to be considered. On the other hand, a declining trend was observed for mental disorders (MNT) and substance use disorders (SBC). An interesting case was the group of neoplasms (NPL), in which it was possible to observe a break in the growing trend in 2017 and then a relatively strong decline.

The following parts of this section focus on evaluating the associations between the share of energy from renewable sources and the health of the population, that is, morbidity of the population in the European Union.

Table 3 presents the test outputs for the assessment of selected assumptions of regression models. As can be seen, the table consists of three sections according to the sectors (transport, electricity, heating and cooling) with four statistical characteristics in each of these sections. Serial correlation was tested using the Baltagi and Li one-sided LM test (BLT), which revealed significant results in all cases, suggesting a more appropriate use of robust estimation methods. Based on the results of the F test for individual effects within countries (F C), it was possible to confirm significant effects in all cases, while the results of the test within years (F Y) indicated a significant effect only in one case (DIA). Hence, it seemed appropriate to use the regression models that take into account the structure of countries, namely a fixed (within) effects model or a random effects model. Given the fact that the effect of years was significant only in one case, a one-way variant of models was preferred in all of the analysed cases, including this individual one. Subsequently, if the robust Hausman test for panel models (RHT) showed a significance at an α level lower than 0.05, the fixed (within) effects model was chosen, otherwise (RHT *p*-value > 0.05) the use of the random effects model was preferred.

Based on the research experience, the authors of this study consider the above-mentioned tests of assumptions to be the best practices and, simultaneously, the minimum requirements necessary for the responsible selection of a suitable model. To ensure relevant results, a very important decision is to choose a model that takes the structure into account or not. Another issue when choosing a model is deciding on model preferences with fixed or random effects. In order to choose an adequate method, it seems reasonable to assess the suitability of using the classical (OLS) model or its robust alternative. However, the robust alternative has the least risk of skewing and disrupting the results, as in the vast majority of cases it does not appear to be detrimental to the results. The previously used tests can responsibly assess all necessary assumptions and support an appropriate and relevant decision.

The outputs in Table 4 present the examined associations between the share of energy from renewable sources in the transport sector and the prevalence of diseases classified into selected diagnosis groups. A significant association could be confirmed in almost all cases, while an exception was observed in models involving chronic respiratory diseases (RSP) and neurological disorders (NRL). Regarding the significant results, the positive β coefficient indicated that in countries where the share of energy from renewable sources in the transport sector was higher, the prevalence of certain types of diseases was also higher. A positive trajectory was observed in most of the analysed diagnosis groups. In contrast, a significant and negative association between the indicators was found in models involving mental disorders (MNT) and substance use disorders (SBC). Thus, in countries with a higher share of energy from renewable sources in the transport sector, a lower prevalence of the mentioned diseases and disorders was observed.

The outputs in Table 5 present the examined associations between the share of energy from renewable sources in the electricity sector and the prevalence of diseases classified into selected diagnosis groups. The results were similar to the previous table; thus, a significant association was not found only in models involving chronic respiratory diseases (RSP) and neurological disorders (NRL). The most significant associations showed positive β coefficients, while the negative ones were identified only in models involving mental disorders (MNT) and substance use disorders (SBC). It was possible to conclude that the outputs were very similar to those in the transport sector and could be interpreted in the same way.

The outputs in Table 6 present the examined associations between the share of energy from renewable sources in the heating and cooling sector and the prevalence of diseases classified into selected diagnosis groups. Again, similar results were as in the previously analysed cases. A different finding was that no significant association was observed only in the model involving chronic respiratory diseases (RSP). Focusing on significant results, the β coefficient indicated positive trajectories in most cases, while negative trajectories were observed in three models, which included mental disorders (MNT), neurological disorders (NRL), and substance use disorders (SBC).

In all three sectoral specifications, the positive trajectories were found for the diagnosis groups such as cardiovascular diseases (CRD), diabetes and kidney diseases (DIA), digestive diseases (DGS), musculoskeletal disorders (MLT), neoplasms (NPL), sense organ diseases (SNS), and skin and subcutaneous diseases (SKN). These associations could be interpreted as meaning that in countries where the share of renewable energy is higher, the prevalence of these diseases is also higher. This finding can be explained by the idea that countries and their main actors are aware of environmental pollution and its negative effects, which are also reflected in the high prevalence of diseases. With more pollution, they take more steps to mitigate these negatives, or do not exacerbate them. Renewable sources provide such a path. When focusing on the coefficients of determination, higher coefficients were observed in the diagnosis groups, such as diabetes and kidney diseases (DIA), musculoskeletal disorders (MLT), sense organ diseases (SNS), and skin and subcutaneous diseases (SKN). The average values (for the observed period 2010–2019) are given in Appendix A. Based on these values, it is possible to assess the examined indicators in comparison between individual countries of the European Union.

Regarding the outputs from a statistical point of view, it was possible to observe the largest deviations when comparing the pooling model with the other models. The outputs of this model, on the one hand, acquired low values of the coefficient of determination and, on the other hand, did not show significant β coefficients compared to the models with fixed and random effects in several cases. On this basis, the fixed or random effects models are more appropriate for estimating the relationships of a data set with a specific structure. There were no pronounced differences between the fixed and random models.

## 4. Discussion

The study as a whole provided some interesting insights into the dimension of health and the dimension of the environment in the European Union. The results of descriptive statistics showed that the average share of renewable energy sources in the electricity sector was 27%, 26% in the heating and cooling sector, and 6% in the transport sector. These results are in line with the information provided in the report on the progress of renewable energy in the European Union, which also indicated that the transport sector is the sector with the lowest share of renewable energy use under the planned trajectory [13]. With a focus on the health indicators, the highest average prevalence of morbidity was found in the diagnosis group of neurological disorders, followed by the group of skin and subcutaneous diseases and digestive diseases. The Member States of the European Union were characterized by the lowest average prevalence of morbidity in the group of substance use disorders.

During the observed period 2010–2019, the share of energy from renewable sources in total energy consumption showed an upward trend. The upward trend was also evident in most diagnosis groups, while the prevalence of mental disorders and substance use disorders showed a declining trend. In recent years, a declining trend in neoplasms has also been identified. Additionally, it was possible to confirm the significant differences in health and environmental indicators between countries. Thus, there was a difference between countries, both in the intensity of the use of renewable energy sources and in the prevalence of diseases.

The results of panel regression models revealed that in countries with a higher share of energy from renewable sources in total energy consumption, the prevalence of diseases such as cardiovascular diseases, diabetes and kidney diseases, digestive diseases, musculoskeletal disorders, neoplasms, sense organ diseases, skin and subcutaneous diseases was also significantly higher. The results of regression analyses mathematically indicated the fact that with a higher share of renewable energy sources, a higher prevalence of the above-mentioned diseases can be expected. However, this interpretation could be misleading, and a higher share of renewable energy sources can be seen as a response to the increasing morbidity, to which environmental pollution has also contributed. The second possible explanation is that some diseases with a high coefficient of determination (diabetes and kidney diseases, musculoskeletal disorders, sense organ diseases, skin and subcutaneous diseases) were frequent in more developed countries, and it can be stated that more developed countries were more inclined to prefer renewable energy than less developed ones in order to reduce poor health. At the same time, changes in health under the influence of the environment manifest themselves later.

These ideas have been supported by other studies. It is well-known that poor health can be the result of environmental degradation [15]. Additionally, the use of non-renewable energy sources can lead to greater environmental degradation and pollution, which have a negative effect on human health [3,4,5]. Therefore, there is a need to focus more on the use of renewable energy, which can have environmental and health benefits [19,20,21]. With high levels of pollution and high levels of poor health, the use of renewable energy sources needs to be considered. At the same time, the savings in pollution-related costs make it possible to increase the level of health care provision, which can be linked to increased health care expenditure and improved public health [16]. This benefit resulting from the use of renewable energy sources can be translated into better diagnostics; therefore, hiding health complications can be detected and treated in time. It is beneficial for the entire population, although increased diagnostics can also lead to increased morbidity rates for a particular period of time due to the higher number of detected diseases. On the other hand, these diseases can be treated in time, and improvements in the health of the whole population could be expected in future. This idea is consistent with the findings of Apergis et al. [16], Mujtaba and Shahzad [17], or Ben Jebli [18], who suggested that higher renewable energy may lead to higher health expenditure and the higher number of doctors. These authors used several statistical methods for panel data in their studies to help reveal the findings, while the methods used in the presented study also expand the knowledge about the problem.

The study also provided a statistical view, and three regression models in a robust variant were used and compared for this purpose. Specifically, it was the OLS pooling model (vcovHC), the fixed effects model (one-way (individual) effect within model), and the random effects model (one-way (individual) Random effect model: Swamy-Arora’s transformation). The most suitable model was selected using tests such as (i) the Baltagi and Li one-sided LM test, (ii) the F test for the presence of individual effects (or time effects), or (iii) the robust regression-based Hausman test. In general, each of the used regression models has its own specifics when considering the data structure and links between individual clusters (in this study, these were countries). From the above-mentioned outputs, it was clear that the pooling model showed different results compared to the fixed and random effects models. These deviations can be explained by the level of acceptance of the data structure. The pooling model acquired low levels of determination coefficients, and simultaneously, the results of this model indicated insignificant results, although the fixed or random effect models supported different results. It should be noted that the applied tests of assumptions strongly recommended a method that takes into account the structure of countries. The structure of the years did not appear in most cases to be a characteristic that should be taken into account. The analytical processes applied in the presented study can be understood as best practice, taking into account computational complexity and added value. The presented analytical processes can relatively reliably capture the relationships in various dimensions, including health and the environment, which have been investigated in similar studies using a much more complex statistical technique [16,17,18]. The panel models have a very wide application for structured data [25] and it can be concluded that very interesting and valuable results can be revealed using the panel models.

Within similar approaches when performing analyses, it is appropriate to use models capable of taking the data structure into account at a sufficient level (e.g., countries). In the absence of this approach, significant bias in results can be expected. This recommendation is especially important for data with shorter time series (e.g., 10 years for annual observations). A potential extension could be the use of models taking into account endogeneity, such as panel models with instrumental variables. In the cases where the structure of countries appears to be significant, the assessment of spatial dependence and the subsequent use of spatial panel models should be considered.

Limitations could also be identified in this research. The prevalence of diseases does not take into account the severity of the disease, and only provides a number. A potential limitation from a statistical point of view may be the fact that data from the last 10 available years have been included in the analyses. For a longer time period, it may be necessary to take into account the time aspect, not just the country aspect. Another limitation that needs to be noted is that all results can only be seen in terms of associations, while a consideration of causal relationships can be misleading.

## 5. Conclusions

The present study examined the associations between the use of renewable energy sources and the prevalence of selected groups of diseases in the European Union. An increased emphasis was placed on the application of analytical methods. It has been revealed that most of the associations showed a significant and positive trajectory. This can be interpreted in several ways suggested in the study. The preference for green energy and the promotion of a sustainable way of life is a trend that is established across the European Union. It should be underlined that green energy is associated with health in several respects, while the results of this study present one of them. At the same time, this issue should be examined in more detail.

Several studies have presented the relationships in given dimensions using a variety of complex analytical techniques, but already using the techniques presented in this study, it is possible to confirm demonstrable results. The application of panel models is the best practice to examine the relationships, when the data are formed by the internal structure of countries (or other spatial clusters) and, simultaneously, a time factor enters the data. Cross-sectional models (e.g., OLS) may not be sufficiently effective in estimations.

The presented study provided a basis for future research, which should focus on a more detailed explanation of the revealed relationships. An interesting view of the issue could be provided by a research that would include individual groups of disease prevalence according to age categories, but also other social, health, and environmental aspects. From a statistical point of view, it would be interesting to evaluate a longer time series, or to assess the appropriateness and relevance of including into the panel models additional instrumental variables, and thus to minimize the problem of endogeneity.

## Figures and Tables

**Table 1:** Descriptive analysis of renewable energy (%) and disease prevalence (per population of 100,000).

Variable	Mean	Median	Std. Dev.	Skew	Kurt	Min	Max	Perc. 25	Perc. 75
RNWe TRP	6.00	5.67	4.68	2.48	8.99	0.00	30.31	3.30	7.30
RNWe ELC	27.18	21.97	18.18	0.78	−0.23	0.03	75.14	12.78	37.52
RNWe H&C	26.35	23.52	15.76	0.58	−0.46	3.10	66.12	14.12	36.22
CRD	11,263.16	11,109.22	2075.40	−0.14	−0.66	6672.92	15,937.50	9785.42	13,076.82
DIA	16,116.13	16,155.27	2286.07	−0.12	−0.41	10,487.50	22,420.39	14,397.74	17,844.75
DGS	28,465.68	28,506.48	4777.42	0.07	−1.40	20,539.97	38,016.59	24,318.52	33,191.05
RSP	9574.34	10,255.49	2652.26	−0.25	−0.26	3745.73	16,411.14	8119.03	11,317.10
MNT	14,041.17	14,285.71	2025.55	0.14	−0.81	10,422.11	18,593.17	12,081.99	15,255.18
MLT	26,272.58	26,910.25	3695.02	0.05	−1.34	19,753.23	33,030.87	22,616.89	29,052.89
NPL	13,721.97	12,895.62	4214.21	0.43	0.77	4805.72	26,139.67	11,254.36	16,303.84
NRL	42,982.91	42,860.85	2262.82	0.64	−0.03	38,629.89	49,080.19	40,907.68	44,329.44
SNS	23,822.02	21,190.89	5908.06	0.33	−1.49	15,039.93	34,449.26	19,084.81	30,143.74
SKN	29,354.82	30,926.95	3988.14	−0.26	−1.72	23,339.69	34,719.56	24,985.81	32,805.99
SBC	3262.79	3283.71	612.95	0.08	−0.58	2011.37	4753.40	2713.95	3677.14

Note: Std. Dev.—standard deviation, Skew—skewness, Kurt—kurtosis, Min—minimum, Max—maximum, Perc. 25—25th percentile, Perc. 75—75th percentile, RNWe TSP—share of energy from renewable sources in transport, RNWe ELC—share of energy from renewable sources in electricity, RNWe H&C—share of energy from renewable sources in heating and cooling, CRD—cardiovascular diseases, DIA—diabetes and kidney diseases, DGS—digestive diseases, RSP—chronic respiratory diseases, MNT—mental disorders, MLT—musculoskeletal disorders, NPL—neoplasms, NRL—neurological disorders, SNS—sense organ diseases, SKN—skin and subcutaneous diseases, SBC—substance use disorders.

**Table 2:** Normality and differences tests—countries and years.

Diff.	RNWe TRP	RNWe ELC	RNWe H&C	CRD	DIA	DGS	RSP	MNT	MLT	NPL	NRL	SNS	SKN	SBC
U SW	0.774 †	0.928 †	0.950 †	0.975 †	0.990 *	0.921 †	0.969 †	0.962 †	0.928 †	0.968 †	0.949 †	0.88 †	0.824 †	0.982 ***
KW C	152.81 †	251.90 †	257.14 †	254.46 †	241.00 †	264.99 †	264.75 †	267.88 †	256.53 †	263.30 †	266.02 †	259.95 †	259.54 †	261.26 †
KW Y	53.52 †	12.24	6.50	10.00	23.30 ***	2.45	0.67	0.10	10.06	0.82	0.13	7.29	8.21	1.49

Significance: * *p*-value < 0.1; *** *p*-value < 0.01; † *p*-value < 0.001. Note: Diff.—differences, U SW—univariate Shapiro-Wilk normality test, KW C—Kruskal Wallis test (countries), KW Y—Kruskal Wallis test (years).

**Table 3:** Assumptions for the selection and application of panel regression models.

Model	Transport				Electricity				Heating and Cooling			
RNWe	BLT	F C	F Y	RHT	BLT	F C	F Y	RHT	BLT	F C	F Y	RHT
CRD	13.58 †	246.5 †	0.63	0.05	13.85 †	324.25 †	0.63	3.44 *	13.43 †	287.66 †	0.49	2.6
DIA	13.08 †	109.12 †	3.71 †	6.92 ***	13.37 †	198.22 †	2.91 ***	29.36 †	13.31 †	136.79 †	2.33 **	15.78 †
DGS	13.2 †	2249.43 †	0.59	12.25 †	13.61 †	3490.59 †	0.06	0.52	13.08 †	3011.69 †	0.02	0.02
RSP	14.5 †	824.2 †	0.24	8.78 ***	14.45 †	827.05 †	0.04	0.9	14.51 †	845.31 †	0.08	0.67
MNT	12.98 †	4206 †	0.02	0.56	13.44 †	4004.27 †	0.06	1.77	13.04 †	4526.27 †	<0.01	<0.01
MLT	12.7 †	566.37 †	0.17	0.65	13.55 †	846.08 †	0.23	0.82	12.82 †	909.62 †	0.62	12 †
NPL	9.53 †	799.46 †	0.02	0.07	9.67 †	663.02 †	0.1	1.9	9.33 †	772.54 †	0.02	0.78
NRL	12.55 †	575.92 †	0.02	2.37	12.48 †	583.6 †	0.01	0.4	12.54 †	540.78 †	0.07	1.46
SNS	13.47 †	867.66 †	1.25	11.74 †	13.8 †	1469.5 †	0.39	4.89 **	13.37 †	1362.03 †	0.13	0.3
SKN	13.05 †	1772.15 †	0.05	6.59 **	13.8 †	3417.79 †	0.05	0.22	13.23 †	2855.85 †	0.25	3.96 **
SBC	12.52 †	582.63 †	0.26	2.78 *	13.59 †	537.66 †	0.44	12.77 †	13.44 †	528.92 †	0.22	3.74 *

Significance: * *p*-value < 0.1; ** *p*-value < 0.05; *** *p*-value < 0.01; † *p*-value < 0.001. Note: RNWe—share of energy from renewable sources in total energy consumption, BLT—Baltagi and Li one-sided LM test, F C—F test for individual effects within countries, F Y—F test for individual effects within years, RHT—robust Hausman test.

**Table 4:** Outputs of PLM models: RNWe transport (%) → prevalence of selected diseases (per population of 100,000).

RNWe	CRD	DIA	DGS	RSP	MNT	MLT	NPL	NRL	SNS	SKN	SBC
**Pooling model**											
β	54.77	−22.62	−214.14 **	162.05 ***	16.77	157.89 **	65.08	48.12	−306.2 ***	261.57 †	7.94
β SE	45.4	39.41	82.83	56.95	35.76	75.81	125.28	36.57	117.31	67.9	12.12
α	10,934.63 †	16,251.82 †	29,750.13 †	8602.36 †	13,940.60 †	25,325.51 †	13,331.60 †	426,94.29 †	25,658.57 †	27,785.87 †	3215.15 †
β SE	588.05	517.49	1170.11	722	525.96	867.31	1068.78	542.03	1609.63	928.29	161.45
R^2^	0.02	0.002	0.04	0.08	0.001	0.04	0.005	0.01	0.06	0.09	0.004
**Fixed (within) effects model**											
β	65.01 ***	128.35 ***	49.70 **	19.95	−8.98 ***	99.64 ***	32.56 *	−6.66	107.69 ***	59.40 ***	−12.85 ***
β SE	24.09	41.86	19.73	12.9	3.34	30.66	17.42	9.28	34.49	17.89	4.58
R^2^	0.17	0.24	0.18	0.04	0.06	0.27	0.04	0.004	0.20	0.27	0.18
**Random effects model**											
β	64.90 †	124.43 †	49.37 †	20.44 †	−8.96 †	99.92 †	32.67 †	−6.4	106.31 †	59.72 †	−12.75 †
β SE	9.16	16.12	7.51	5.8	2.52	12.36	7.41	4.28	14.06	7.47	2.72
α	10,873.9 †	15,369.8 †	28,169.5 †	9451.8 †	14,094.9 †	25,673.2 †	13,526 †	43,021.3 †	23,184.4 †	28,996.6 †	3339.3 †
β SE	405.44	435.53	913.35	495.96	401.42	715.7	830.03	443.7	1108.61	743.34	120.47
R^2^	0.16	0.21	0.16	0.04	0.06	0.27	0.25	0.004	0.18	0.25	0.16

Significance: * *p*-value < 0.1, ** *p*-value < 0.05, *** *p*-value < 0.01, † *p*-value < 0.001. Note: SE—standard error. The preferred model is underlined.

**Table 5:** Outputs of PLM models: RNWe electricity (%) → prevalence of selected diseases (per population of 100,000).

	CRD	DIA	DGS	RSP	MNT	MLT	NPL	NRL	SNS	SKN	SBC
**Pooling model**											
β	22.55	3.35	21.47	28.93	18.95	55.40 *	90.61 *	−5.35	−15.96	40.9	8.70 *
β SE	17.05	19.38	44.63	26.41	16.85	31.6	52.38	16.35	59.16	34.01	4.49
α	10,650.25 †	16,025.21 †	27,882.16 †	87,88.09 †	13,526.26 †	24,767.01 †	11,259.57 †	43,128.37 †	24,255.81 †	28,243.31 †	3026.39 †
β SE	717.83	733.62	1612.42	860.36	597.14	1088.19	1412.77	675.06	2046.22	1202.94	191.36
R^2^	0.04	<0.001	0.007	0.04	0.03	0.07	0.15	0.002	0.002	0.03	0.07
**Fixed (within) effects model**											
β	63.41 †	130.52 †	52.34 †	−0.79	−4.92	92.19 †	13.49	6.45	111.01 †	58.50 †	−8.20 ***
β SE	17.38	21.85	11.9	10.37	3.26	18.36	14.89	10.34	21.82	9.7	2.6
R^2^	0.38	0.56	0.45	<0.001	0.04	0.52	0.02	0.01	0.5	0.59	0.17
**Random effects model**											
β	61.25 †	119.33 †	52.18 †	−0.16	−4.81 ***	91.43 †	15.53 **	6.1	109.47 †	58.41 †	−7.65 †
β SE	5.96	7.71	4.25	4.36	1.49	6.43	7.15	4.02	7.69	3.52	1.23
α	9598.6 †	12,873.4 †	27,047.7 †	9578.6 †	14,171.9 †	23,788 †	13,299.9 †	42,817.1 †	20,847.1 †	27,767.6 †	3470.6 †
β SE	428.56	501.13	951.23	524.69	398.34	722.32	778.09	454.95	1190	782.44	118.48
R^2^	0.35	0.49	0.42	<0.001	0.04	0.5	0.02	0.01	0.47	0.57	0.14

Significance: * *p*-value < 0.1, ** *p*-value < 0.05, *** *p*-value < 0.01, † *p*-value < 0.001. Note: SE—standard error. The preferred model is underlined.

**Table 6:** Outputs of PLM models: RNWe heating and cooling (%) → prevalence of selected diseases (per population of 100,000).

	CRD	DIA	DGS	RSP	MNT	MLT	NPL	NRL	SNS	SKN	SBC
**Pooling model**											
β	56.40 ***	44.57 *	74.38	−27.32	−11.31	−10.76	71.38 *	−39.02	103.04	−25.96	4.45
β SE	17.28	23.36	62.63	39.34	18.58	34.61	37.44	23.94	81.43	47.49	6.2
α	9777.02 †	149,41.62 †	26,505.66 †	10,294.15 †	14,339.30 †	26,556.14 †	11,841.08 †	44,011.15 †	21,106.74 †	30,038.90 †	31,45.52 †
β SE	612.38	783.77	1778.32	920.17	686.17	1149.22	1147.23	862.29	1976.52	1346.15	241.13
R^2^	0.18	0.09	0.06	0.03	0.01	0.002	0.07	0.07	0.08	0.01	0.01
**Fixed (within) effects model**											
β	86.59 †	151.71 †	64.75 †	7.8	−11.33 ***	121.30 †	38.48 ***	−8.48	146.35 †	70.87 †	−8.57 ***
β SE	13.15	17.93	8.47	12.32	3.95	12.86	13.13	8.02	15.21	8.01	2.6
R^2^	0.41	0.44	0.39	0.01	0.14	0.52	0.07	0.01	0.50	0.50	0.01
**Random effects model**											
β	84.31 †	135.63 †	64.82 †	6.85	−11.33 †	117.95 †	39.44 †	−9.75 ***	145.62 †	70.08 †	−8.01 †
β SE	5.8	8.25	4.23	5.02	1.78	6.45	8.47	3.58	8.02	3.86	1.51
α	9041.4 †	12,542.1 †	26,757.5 †	93,93.8 †	14,339.7 †	23,164.3 †	12,682.5 †	43,239.8 †	19,984.7 †	27,508.1 †	3473.9 †
β SE	397.86	500.55	928.01	535.03	403.37	760.89	837.01	444.14	1145.72	798.11	121.16
R^2^	0.39	0.39	0.37	0.01	0.13	0.48	0.07	0.01	0.48	0.47	0.09

Significance: * *p*-value < 0.1, *** *p*-value < 0.01, † *p*-value < 0.001. Note: SE—standard error. The preferred model is underlined.

## Data Availability

The analytical procedures included data from the Eurostat database, namely the share of energy from renewable sources as an environmental indicator, and data from the Global Burden of Disease Study, specifically, health indicators of disease prevalence. The data were collected for the period 2010–2019. Thus, each of the countries of the European Union reported annual data for the observed period, that is, 10 years for individual variables.

## Appendix A

**Table A1:** Mean values of selected variables (2010–2019).

Country	TRP	ELC	H&C	CRD	DIA	DGS	RSP	MNT	MLT	NPL	NRL	SNS	SKN	SBC
Austria	10.29 (25)	70.55 (27)	33.05 (18)	11,995.34 (15)	14,604.57 (7)	28,605.03 (15)	10,891.02 (19)	15,072.71 (19)	30,145.21 (22)	25,289.11 (27)	42,936.21 (27)	19,614.34 (11)	32,698.34 (21)	3876.79 (21)
Belgium	5.55 (15)	14.21 (9)	7.66 (4)	10,144.64 (9)	14,370.78 (6)	24,354.64 (7)	11,159.69 (20)	14,263.05 (14)	28,854.01 (20)	12,116.78 (12)	46,437.59 (12)	19,352.43 (9)	32,427.43 (18)	3341.99 (18)
Bulgaria	5.16 (11)	18.12 (11)	29.17 (17)	13,697.59 (23)	18,339.94 (23)	33,317.98 (20)	7943.18 (7)	11,647.45 (5)	22,935.61 (10)	15,030.29 (19)	40,760.05 (19)	31,876.83 (25)	25,204.74 (9)	2533.64 (9)
Croatia	2.18 (4)	43.76 (22)	36.31 (20)	12,224.86 (19)	18,526.16 (26)	33,378.37 (22)	8631.18 (9)	12,131.5 (8)	22,762.95 (9)	19,655.13 (26)	39,401.37 (26)	30,825.48 (23)	25,061.99 (7)	3272.17 (7)
Cyprus	1.95 (2)	6.89 (3)	25.33 (14)	6896.15 (1)	13,733 (5)	22,108.02 (3)	10,517.64 (16)	15,264.53 (21)	27,320.22 (14)	6771.34 (2)	44,643.92 (2)	15,904.68 (2)	30,409.21 (13)	2447.41 (13)
Czechia	6.02 (18)	12.56 (7)	18.56 (11)	13,736.73 (24)	20,405.19 (27)	31,239.39 (17)	6853.13 (5)	11,411.57 (3)	21,725.74 (2)	16,077.45 (20)	40,510.88 (20)	29,914.87 (20)	24,856.08 (6)	3580.22 (6)
Denmark	5.82 (16)	49.16 (23)	38.88 (22)	10,441.61 (12)	13,491.52 (3)	24,446.43 (8)	12,074.19 (24)	13,545.63 (11)	31,929.56 (26)	11,481.98 (9)	43,231.79 (9)	18,830.5 (6)	33,338.27 (23)	4086.96 (23)
Estonia	1.19 (1)	15.5 (10)	47.61 (24)	14,233.23 (26)	18,490.17 (25)	31,978.44 (18)	4029.94 (1)	12,661.53 (9)	22,758.09 (8)	17,399.96 (23)	41,308.86 (23)	31,842.21 (24)	25,583.48 (11)	3632.14 (11)
Finland	13.32 (26)	32.43 (19)	51.37 (26)	11,061.36 (14)	16,361.67 (13)	25,192.62 (11)	10,429.28 (15)	14,190.57 (13)	28,509.64 (19)	12,938.3 (14)	43,335.64 (14)	19,282.2 (8)	33,727.19 (24)	3200.79 (24)
France	7.46 (24)	18.45 (12)	18.79 (12)	9738.38 (8)	11,922.49 (2)	21,669.23 (2)	10,270.95 (14)	15,819.77 (23)	27,545.47 (17)	11,142.53 (7)	41,250.06 (7)	19,198.79 (7)	34,210.18 (27)	3087.08 (27)
Germany	7.06 (23)	29.26 (16)	13.34 (6)	12,064.65 (16)	18,414.5 (24)	24,750.61 (10)	10,710.45 (17)	14,942.95 (18)	31,299.8 (24)	16,627.29 (22)	45,920.16 (22)	21,094.11 (14)	33,913.29 (26)	2996.47 (26)
Greece	2.06 (3)	21.22 (14)	25.89 (15)	10,189.7 (10)	17,507.1 (18)	28,100.84 (13)	10,757.56 (18)	16,783.72 (25)	28,874.22 (21)	11,591.11 (10)	44,703.94 (10)	20,570.22 (12)	32,603.84 (19)	2135.66 (19)
Hungary	7.01 (22)	7.39 (4)	20.49 (13)	13,852.71 (25)	18,167.67 (22)	33,366.58 (21)	8743.44 (11)	11,765.98 (6)	22,454.73 (7)	14,946.49 (18)	40,901.37 (18)	30,423.7 (21)	25,363.15 (10)	3248.81 (10)
Ireland	5.51 (14)	25.07 (15)	5.69 (2)	7419.75 (2)	11,678.05 (1)	23,053.83 (4)	11,497.48 (21)	16,507.88 (24)	26,185.55 (12)	9868.5 (4)	42,869.83 (4)	15,822.57 (1)	30,922.45 (14)	4546.18 (14)
Italy	6.37 (19)	30.63 (17)	18.07 (10)	14,605.79 (27)	17,594.72 (19)	37,189.69 (27)	8679.5 (10)	14,918.43 (17)	31,964.41 (27)	14,699.52 (15)	48,421.92 (15)	22,865.69 (16)	33,898.12 (25)	2331.32 (25)
Latvia	3.84 (7)	49.61 (24)	50.59 (25)	13,462.54 (22)	18,061.37 (20)	33,816.97 (25)	5195.26 (3)	13,086.07 (10)	23,619.18 (11)	14,793.1 (17)	41,500.43 (17)	33,262.08 (27)	23,841.07 (1)	3746.29 (1)
Lithuania	4.27 (8)	14.2 (8)	40.99 (23)	13,121.49 (21)	16,532.94 (16)	35,096.71 (26)	5185.32 (2)	13,914.82 (12)	22,387.69 (6)	18,524.49 (24)	42,148.49 (24)	32,768.92 (26)	24,243.8 (3)	3255.09 (3)
Luxembourg	5.03 (10)	6.47 (2)	6.54 (3)	9217.82 (4)	14,643.05 (8)	23,729.28 (6)	11,776.49 (23)	14,297.1 (15)	27,602.47 (18)	10,895.59 (6)	42,905.67 (6)	17,278.69 (3)	32,038.12 (15)	3662.82 (15)
Malta	4.69 (9)	3.91 (1)	16.3 (9)	9585.12 (6)	17,201.48 (17)	24,551.19 (9)	11,504.91 (22)	15,086.4 (20)	30,290.01 (23)	10,457.97 (5)	44,089.29 (5)	19,585.42 (10)	32,166.74 (16)	2609.41 (16)
Netherlands	6.41 (20)	12.03 (6)	4.86 (1)	9676.29 (7)	13,603.42 (4)	21,142.63 (1)	13,009.57 (26)	15,779.11 (22)	27,541.39 (16)	11,914.72 (11)	47,478.07 (11)	18,701.03 (5)	32,186.42 (17)	2488.42 (17)
Poland	5.87 (17)	11.59 (5)	14.28 (7)	10,497.41 (13)	16,417.41 (14)	32,477.56 (19)	8973.15 (12)	10,495.84 (1)	21,942.91 (3)	4888.39 (1)	42,166.8 (1)	27,431.59 (18)	24,335.36 (5)	4330.99 (5)
Portugal	5.28 (13)	50.18 (25)	38.26 (21)	9457.32 (5)	18,166.48 (21)	28,273.99 (14)	15,246.19 (27)	18,308.74 (27)	31,825.95 (25)	9101.54 (3)	44,412.49 (3)	20,602.03 (13)	33,252.74 (22)	3853.25 (22)
Romania	5.17 (12)	38.56 (21)	26.07 (16)	12,205.75 (18)	14,726.88 (9)	33,497.28 (23)	7808.37 (6)	11,385.9 (2)	21,975.04 (5)	19,010.53 (25)	40,405.36 (25)	29,649.48 (19)	24,267.89 (4)	2659.77 (4)
Slovakia	6.94 (21)	21.08 (13)	10.35 (5)	10,428.26 (11)	14,888.72 (10)	30,573.99 (16)	5408.45 (4)	11,525.33 (4)	20,582.25 (1)	16,428.26 (21)	40,724.37 (21)	27,350.11 (17)	23,913.71 (2)	3303.54 (2)
Slovenia	3.54 (6)	32.41 (18)	33.51 (19)	12,780.51 (20)	16,448.28 (15)	33,544.51 (24)	8197.59 (8)	12,053.46 (7)	21,952.81 (4)	14,774.57 (16)	40,620.63 (16)	30,496.62 (22)	25,204.55 (8)	3609.95 (8)
Spain	3.53 (5)	35.1 (20)	15.88 (8)	9187.23 (3)	15,585.58 (12)	25,854.32 (12)	10,120.65 (13)	17,466.43 (26)	27,503.14 (15)	11,260.46 (8)	43,761.2 (8)	21,203.97 (15)	30,284.94 (12)	2911.2 (12)
Sweden	20.44 (27)	63.4 (26)	63.68 (27)	12,183.19 (17)	15,252.46 (11)	23,263.14 (5)	12,892.57 (25)	14,785.14 (16)	26,871.48 (13)	12,807.88 (13)	43,692.26 (13)	17,445.93 (4)	32,627.15 (20)	3346.99 (20)

Note: The number in parentheses represents the order—the lowest value is marked (1) and the highest (27).

## References

[1] European Commission Renewable Energy Directive. https://ec.europa.eu/energy/topics/renewable-energy/renewable-energy-directive/overview_en.

[2] Panwar N.L., Kaushik S.C., Kothari S. (2011). Role of renewable energy sources in environmental protection: A review. Renew. Sust. Energ. Rev..

[3] Koornneef J., Van Harmelen T., Van Horssen A., Ramirez A., Mazzeo N. (2011). Carbon dioxide capture and air quality. Chemistry, Emission Control, Radioactive Pollution and Indoor Air Quality.

[4] Dilmore R., Zhang L., Romanov V. (2018). Greenhouse gases and their role in climate change. Greenhouse Gases and Clay Minerals. Green En-ergy and Technology.

[5] McMichael A.J., Woodruff R.E., Hales S. (2006). Climate change and human health: Present and future risks. Lancet.

[6] Amarante J.C.A., Besarria C.N., de Souza H.G., dos Anjos O.R. (2021). The relationship between economic growth, renewable and nonrenewable energy use and CO2 emissions: Empirical evidences for Brazil. Greenh. Gas. Sci. Technol..

[7] Omri A., Belaid F. (2021). Does renewable energy modulate the negative effect of environmental issues on the socio-economic welfare?. J. Environ. Manag..

[8] European Commission A European Green Deal. https://ec.europa.eu/info/strategy/priorities-2019-2024/european-green-deal_en.

[9] Manopriya S., Hareesh K. (2021). The prospects and challenges of solar electrochemical capacitors. J. Energy Storage.

[10] European Commission Renewable Energy. https://ec.europa.eu/energy/topics/renewable-energy_en.

[11] Annibaldi V., Condemi A., Cucchiella F., Gastaldi M., Rotilio M. (2020). Renewable energy policies: Bibliometric review and policy implications. Environ. Clim. Technol..

[12] European Commission (2020). Report from the Commission to the European Parliament, the Council, the European Economic and Social Committee and the Committee of the Regions. Renewable Energy Progress Report.

[13] European Commission (2020). Technical Assistance in Realisation of the 5th Report on Progress of Renewable Energy in the EU. Task 1-2: Final Report.

[14] Kousar S., Ahmed F., López García M.D.L.N., Ashraf N. (2020). Renewable energy consumption, water crises, and environmental degradation with moderating role of governance: Dynamic panel analysis under cross-sectional dependence. Sustainability.

[15] Lu Z.N., Chen H., Hao Y., Wang J., Song X., Mok T.M. (2017). The dynamic relationship between environmental pollution, economic development and public health: Evidence from China. J. Clean. Prod..

[16] Apergis N., Ben Jebli M., Ben Youssef S. (2018). Does renewable energy consumption and health expenditures decrease carbon dioxide emissions? Evidence for sub-Saharan Africa countries. Renew. Energ..

[17] Mujtaba G., Shahzad S.J.H. (2021). Air pollutants, economic growth and public health: Implications for sustainable development in OECD countries. Environ. Sci. Pollut. Res..

[18] Ben Jebli M. (2016). On the causal links between health indicator, output, combustible renewables and waste consumption, rail transport, and CO_2_ emissions: The case of Tunisia. Environ. Sci. Pollut. Res..

[19] Taghizadeh-Hesary F., Rasoulinezhad E., Yoshino N., Chang Y., Taghizadeh-Hesary F., Morgan P.J. (2021). The energy–pollution–health nexus: A panel data analysis of low- and middle-income Asian countries. Singapore Econ. Rev..

[20] Khan S.A.R., Godil D.I., Quddoos M.U., Yu Z., Akhtar M.H., Liang Z. (2021). Investigating the nexus between energy, economic growth, and environmental quality: A road map for the sustainable development. Sustain. Dev..

[21] Wang Q., Li L., Zhang Y., Cui Q., Fu Y., Shi W., Wang Q., Xu D. (2021). Research on the establishment and application of the environmental health indicator system of atmospheric pollution in China. Bull. Environ. Contam. Toxicol..

[22] Buonocore J.J., Luckow P., Norris G., Spengler J.D., Biewald B., Fisher J., Levy J.I. (2015). Health and climate benefits of different energy-efficiency and renewable energy choices. Nat. Clim. Chang..

[23] Buonocore J.J., Hughes E.J., Michanowicz D.R., Heo J., Allen J.G., Williams A. (2019). Climate and health benefits of increasing renewable energy deployment in the United States. Environ. Res. Lett..

[24] Baltagi B.H., Egger P., Pfaffermayr M. (2013). A generalized spatial panel data model with random effects. Econ. Rev..

[25] Croissant Y., Giovanni M. (2019). Panel Data Econometrics with R, United States.

[26] Breusch T.S., Pagan A.R. (1979). A simple test for heteroscedasticity and random coefficient variation. Econometrica.

[27] Wooldridge J.M. (2010). Econometric Analysis of Cross–Section and Panel Data.

[28] Baltagi B.H., Li Q. (1995). Testing AR(1) against MA(1) disturbances in an error component model. J. Econ..

[29] Angrist J.D., Newey W.K. (1991). Over-identification tests in earnings functions with fixed effects. J. Bus. Econ. Stat..

[30] Danovi A., Olgiati S., D’Amico A. (2021). Living longer with disability: Economic implications for healthcare sustainability. Sustainability.

[31] Sopko J., Kočišová K. (2019). Key indicators and determinants in the context of the financial aspects of health systems in selected countries. Adiktologie.

[32] Androniceanu A.M., Georgescu I., Dobrin C., Dragulanescu I.V. (2020). Multifactorial components analysis of the renewable energy sector in the OECD countries and managerial implications. Pol. J. Manag. Stud..

[33] Ahmed U., AlZgool M.R.H., Shah S.M.M. (2020). The impact of green human resource practices on environmental performance. Pol. J. Manag. Stud..

[34] Štreimikienė D. (2021). Externalities of power generation in Visegrad countries and their integration through support of renewables. Econ. Soc..

[35] Flessa S., Meissner K. (2019). Sustainability of health systems research—A conceptional framework based on two projects. Econ. Soc..

[36] Gavurova B., Toth P., Ciutienė R., Tarhanicova M. (2019). The impact of healthcare availability on the amenable mortality: Country study. Econ. Soc..

[37] Zhou M., Liao J., Hu N., Kuang L. (2020). Association between primary healthcare and medical expenditures in a context of hospital-oriented healthcare system in China: A national panel dataset, 2012–2016. Int. J. Environ. Res. Public Health.

[38] Baptista E.A., Kakinuma K., Queiroz B.L. (2020). Association between cardiovascular mortality and economic development: A spatio-temporal study for prefectures in Japan. Int. J. Environ. Res. Public Health.

[39] Gavurová B., Kováč V., Vagašová T. (2017). Standardised mortality rate for cerebrovascular diseases in the Slovak Republic from 1996 to 2013 in the context of income inequalities and its international comparison. Health Econ. Rev..

[40] Gavurová B., Vagašová T. (2018). Potential gains in life expectancy by eliminating deaths from cardiovascular diseases and diabetes mellitus in the working life ages among Slovak population. Health Econ. Rev..

[41] Simionescu M., Bilan S., Gavurova B., Bordea E.N. (2019). Health policies in Romania to reduce the mortality caused by cardiovascular diseases. Int. J. Environ. Res. Public Health.

[42] Eurostat Eurostat Database. https://ec.europa.eu/eurostat/data/database.

[43] (2019). Global Burden of Disease Study. Results. 2019.

